# Mixed comparison of interventions for different exercise types on students with Internet addiction: a network meta-analysis

**DOI:** 10.3389/fpsyg.2023.1111195

**Published:** 2023-05-25

**Authors:** Yihan Zhang, Geng Li, Chengzhen Liu, Huohong Chen, Jianping Guo, Zifu Shi

**Affiliations:** ^1^School of Educational Science, Hunan Normal University, Changsha, China; ^2^School of Physical Education, Hunan Normal University, Changsha, China; ^3^Key Laboratory of Sports Intelligence Research, Hunan Normal University, Changsha, China; ^4^Cognition and Human Behavior Key Laboratory of Hunan Province, Changsha, China

**Keywords:** student, internet addiction, mental health, physical activity, treatment

## Abstract

**Background:**

Internet addiction (IA) has a significant negative impact on students. The condition of students with IA can be improved by exercise, which has been identified as an effective intervention strategy. However, the relative effectiveness of different exercise types and the most effective ones remains unknown. This study presents a network meta-analysis to compare six exercise types (team sport, double sport, single sport, team + double sport, team + single sport, and team + double + single sport) based on their effectiveness in reducing Internet addiction and maintaining mental health.

**Methods:**

Systematic searches were conducted in PubMed, EMBASE, Cochrane Library, CNKI, Wan Fang, CQVIP, Web of Science, CBM, EBSCO, APA PsycNet, and Scopus, and all relevant studies from the beginning to 15 July 2022 were included on. According to the Cochrane Handbook 5.1.0 Methodological Quality Evaluation Criteria, the listed studies' bias risk was assessed, while the network meta-analysis was performed using STATA 16.0.

**Results:**

A total of 39 randomized controlled trials that met all inclusion criteria including 2,408 students with IA were examined. The meta-analysis results showed that compared with the control group, exercising significantly improved loneliness, anxiety, depression, and interpersonal sensitivity (*p* < 0.05). Specifically, the network meta-analysis discovered that the single sport, team sport, double sport, team + double sport, and team + double + single sport had significant effects on improving Internet addiction as compared to the respective control group (*p* < 0.05); the single sport, team sport, and double sport tend to be effective compared with controls in improving mental health (*p* < 0.05). Compared with the other five types of sports, the double sport was ranked first and showed the greatest potential to be the best choice (cluster ranking value = 3699.73) in improving Internet addiction (SUCRA = 85.5) and mental health (SUCRA = 93.1).

**Conclusion:**

Exercise could be suggested as the best alternative when treating IA in students, based on the extensive positive effects of exercise on IA, anxiety, depression, interpersonal sensitivity, loneliness, and mental health in IA students. Double sport may be the best type of exercise for Internet-addicted students. However, to further examine the benefits of exercise for IA students, more research is required.

**Systematic review registration:**

https://www.crd.york.ac.uk/PROSPERO/display_record.php?RecordID=377035, identifier: CRD42022377035.

## 1. Introduction

Internet addiction (IA) has become a serious physical and mental health problem among students (Hu and Ma, 2010). Internet addiction is defined as the inability to control the use of the Internet and is a condition that leads to severe impairments of various life functions (Young and Rogers, 1998; Griffiths, 1999; Mok et al., 2014). With the popularity of smartphones and computers, students have become a high-risk group for IA, with the prevalence ranging from 0.8 to 26.7% in different countries or regions (Kuss et al., 2014; Ma et al., 2022a). IA endangers students' physical and mental health and academic performance by triggering a series of mental illnesses, crimes, and self-harming behaviors (Gentile et al., 2011; Lam, 2014; Spada, 2014; Fang et al., 2015). Especially since the outbreak of COVID-19, closed living environments and online courses have increased the use of electronic devices such as mobile phones, resulting in a continued increase in students with IA (Li et al., 2021; Shehata and Abdeldaim, 2021; Besalti and Satici, 2022). Psycho-psychotherapy and pharmacotherapy are two common methods used to treat IA (Shao et al., 2020; Zhai et al., 2020; Zhang Z. et al., 2022). However, due to the limitations of psycho-psychotherapy and pharmacotherapy such as stigma, long cycle time, high cost, and side effects, students often refuse treatment (Ma et al., 2022b). As a result, both treatments are typically slower and less desirable (Sun et al., 2010). Therefore, there is an urgent need to find a more scientifically effective and easier-to-implement intervention method.

Due to its simplicity and effectiveness, exercise is widely accepted among students as a way to improve physical function (Bu et al., 2010). In recent years, studies have suggested that exercise can effectively reduce IA (Li et al., 2009; Yu and Xie, 2010; Li M. et al., 2014; Park et al., 2016; Wu et al., 2019; Liu et al., 2022). Researchers found that running, as a single sport, can effectively reduce anxiety and depression, as well as IA symptoms (Gordon et al., 1986; Nicholson et al., 2011; Oriel et al., 2011). Additionally, double sport with certain interactivity and avoiding physical collision is more suitable for IA students who have poor fitness due to long-term Internet addiction to alleviate IA by alleviating loneliness (Gao and Chen, 2006; Zhang et al., 2015; Li et al., 2022). Moreover, team sports, and mixed sports as alternating different sports types, are thought to reduce IA by relieving interpersonal sensitivity (Wang, 2016; Yang, 2021). From the above evidence, different types of exercise based on respective psychological benefits can effectively reduce IA (Miller et al., 2020). The relationship between participants during the intervention has been considered a factor for IA (Yu et al., 2021). Therefore, from an interpersonal perspective, it is reasonable to hypothesize that there are relatively large differences in the anti-internet addiction effects of different exercise types. Even though researchers have conducted correlational studies, randomized controlled trials, and meta-analyses on exercise interventions for IA students (Wu et al., 2018, 2019; Liu et al., 2019; Qiao et al., 2020; Zhang Z. et al., 2022), no study has comprehensively compared the effects of different exercise types on IA students, nor has it proposed the optimal exercise type to help IA students.

Network meta-analysis (NMA) is considered a useful method to compare more than two interventions (Lumley, 2002; Jansen and Naci, 2013; Li L. et al., 2014). Meanwhile, NMA allows the use of indirect comparison methods to quantitatively compare different interventions for the treatment of similar conditions and thus select the best treatment regimen (Bucher et al., 1997; Tian et al., 2013). We used NMA to investigate the differential effects of different exercise types on IA students. Moreover, mental health is a key factor in IA (Ko et al., 2012; Babadi-Akashe et al., 2014). IA students scored higher on their loneliness, anxiety, depression, and interpersonal sensitivity than healthy controls on subjective (Long et al., 2021; Zhang et al., 2021; Qiu et al., 2022; Yang et al., 2022). Thus, treatment should not only focus on whether the level of IA is decreased but also on the improvement of mental health indicators related to IA (Li et al., 2017; Zhang et al., 2018). We integrated the effects of interventions on IA and mental health and explore the utility of the interventions and relevant intervention mechanisms. We used the two-dimensional clustered ranking map of NMA to comprehensively rank the effects of different exercise types and identified the best treatment for IA students.

Accordingly, the goals of this network meta-analysis were to (1) quantitatively compare the effect of different exercise types for Internet-addicted students by comprehensively considering the improvement of IA and mental health; and (2) screen out the most suitable intervention project for IA students by the comprehensive evaluation and ranking of the effects of different sports types. Specifically, we hypothesized that (1) the exercise intervention would have positive effects on loneliness (H1a), anxiety (H1b), depression (H1c), and interpersonal sensitivity (H1d) on IA students; (2) the double sport with some interaction would have the best intervention effects for Internet addiction (H2a) and mental health (H2b); and (3) the double sport would be the optimal exercise type under the combined consideration of IA and mental health (H3).

## 2. Methods

The Preferred Reporting Items for Systematic Reviews and Meta-Analyses statement extension for systematic reviews incorporating network meta-analysis (PRISMA-NMA) was used to report this systematic review and network meta-analysis (Hutton et al., 2016). This review was registered with the International Prospective Register of Systematic Review (PROSPERO) (CRD42022377035).

### 2.1. Search strategy

The databases of Web of Science, CNKI, Cochrane Library, EBSCO, EMBASE, Wan fang, CQVIP, Scopus, PubMed, CBM, and APA PsycNet were searched from the beginning to 15 July 2022. Search terms included “exercises” and “internet addiction” along with numerous other related terms. The language of the literature was not limited to the searches. The full search strategies are detailed in the Supplementary material.

### 2.2. Eligibility criteria

Inclusion and exclusion criteria for the literature were (1) inclusion of studies related to students with IA and exclusion of studies about animals, elderly, and special population groups; (2) inclusion of randomized controlled trials (RCT) and exclusion of review literature, review literature, systematic evaluation literature, and qualitative studies; (3) inclusion of studies related to exercise interventions and exclusion of studies combining other interventions; (4) inclusion of studies where reporting data can be integrated; and (5) inclusion of different forms of IA with similar effects (Young, 1999; Chi and Chiu, 2013).

### 2.3. Exercise types

Seven types were designed according to the number of participants and the interrelationship of participants in the exercise to classify the interventions of the included studies (Han et al., 2014), along with a control team was used in this research:

1. Team Sport: A team is made up of multiplayer who work together in a planned manner, and the competitive contest between the team and the team is carried out according to the corresponding activity rules. The overall level is determined by their cooperation rather than individual ability, such as in basketball and soccer.
2. Double Sport: A competitive sport in which two people compete against each other, such as table tennis, badminton, and tennis.
3. Single Sport: A sport of a single person is mainly individual performance, with skill leading performance difficulty and physical ability leading performance items, such as Ba Duan Jin, Tai Chi, and yoga.
4. Team + Double Sport: The combined exercise arrangement of team sport and double sport, such as badminton + basketball.
5. Team + Single Sport: The combined exercise arrangement of team sport and single sport, such as badminton + running.
6. Team + Double + Single Sport: The combined exercise arrangement of team sport, double sport, and individual sport, such as basketball + badminton + running.
7. No intervention; Control team.

### 2.4. Study selection and data extraction

Two researchers (Geng LI and Chenzhen LIU) independently worked according to the pre-developed literature screening criteria. First, NoteExpress software was used to find duplicate title information. Second, two researchers evaluated the potential eligibility of each abstract generated by the search strategy, and the full text of the research will be available unless both reviewers determine that an abstract is ineligible. Third, each full-text report was independently evaluated for final study inclusion.

We extracted the following data from various studies: (1) sample size; (2) year of publication; (3) first author; (4) sample source; (5) outcome indicators; (6) intervention type; (7) intervention duration; (8) exercise intensity; (9) gender and age of participants; (10) location and language of study; (11) frequency of a week; and (12) length of a session. The above information was extracted using a pre-developed form, and the extracted data were cross-checked by two researchers. Disagreements regarding the inclusion of extracted information were decided by the third researcher (Zifu SHI).

### 2.5. Risk of bias assessment

The risk of bias was independently assessed according to the Cochrane Handbook 5.1.0 Methodological Quality Evaluation Criteria (Higgins et al., 2011) by two researchers. The relevant indicators in this assessment were divided into random sequence method, allocation concealment, blinding by researchers and subjects, selective reporting, completeness of outcome data, and other biases. Different colors (green, red, and yellow) represented the judgment of “low-risk bias”, “high-risk bias”, and “unclear”. Every disagreement was discussed and finally was decided by the third researcher.

### 2.6. Statistical analysis

The network meta-analysis based on the frequency-based framework was performed by Stata 16.0. Means and standard deviations as outcome indicators were used to compare the effects of different exercise interventions on IA students. Among the outcome indicators of this study, IA, loneliness, and anxiety were measured by different questionnaires. To combine the effect sizes, the standardized mean difference (SMD) was used (Qiao et al., 2020). Mental health, depression, and interpersonal sensitivity were measured by the same questionnaire. Thus, the weighted mean difference (WMD) was used to combine the effect sizes. The inconsistency test was performed using nodal analysis. If the difference between direct and indirect comparisons was not statistically significant (*P* > 0.05), the consistency model was selected for analysis; conversely, the inconsistency model was selected (Fei et al., 2022). The surface under the cumulative ranking probability plot (SUCRA) was selected to compare and rank the effects of different exercise types. In the SUCRA, the larger the area under the curve, the greater the likelihood of being the best intervention (Salanti et al., 2011). Based on the SUCRA of different exercise types in IA and mental health, the two-dimensional clustered ranking map was constructed to determine the best treatment choice.

## 3. Results

### 3.1. Literature selection

**Figure 1:**
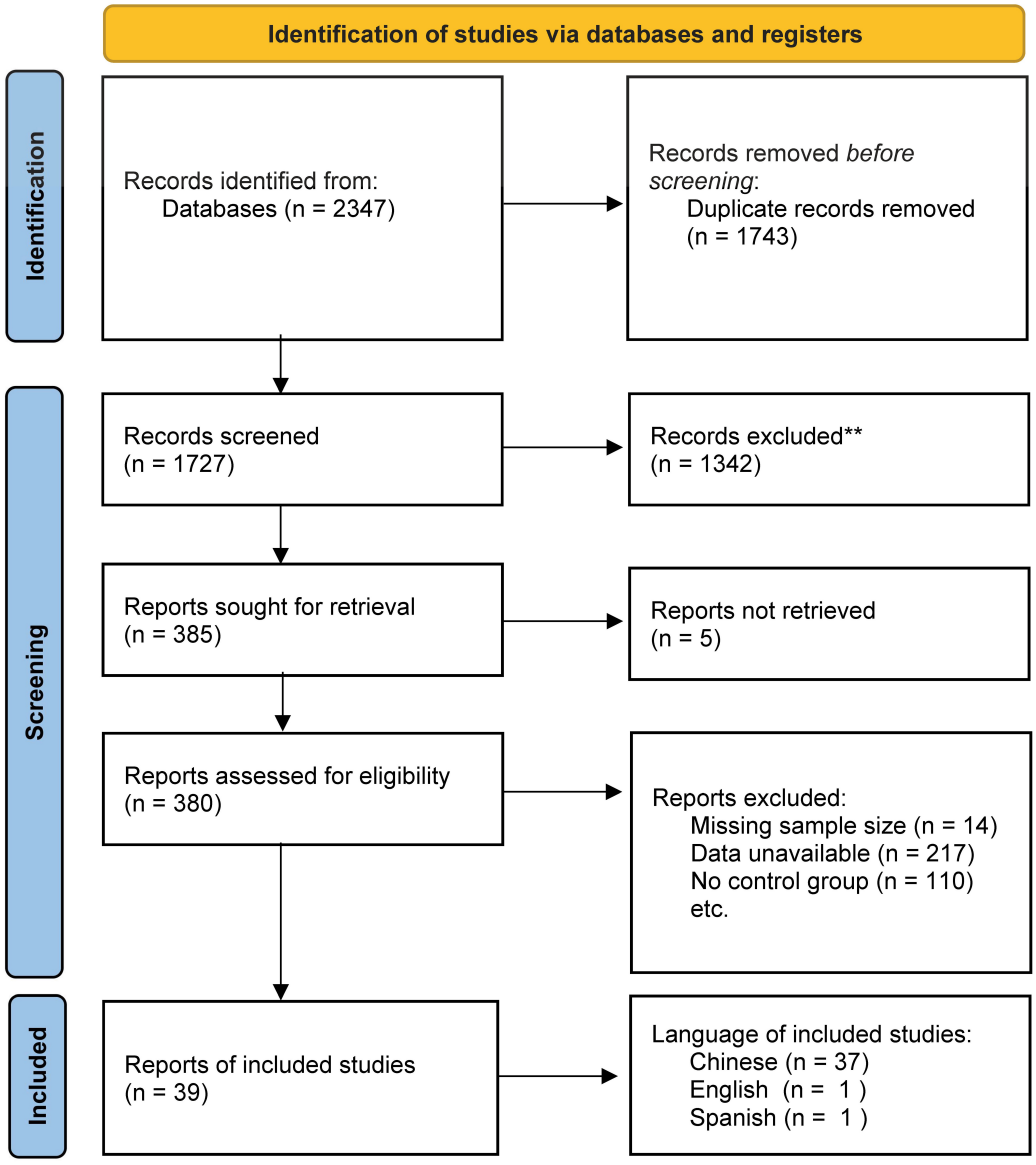
Flowchart of screening process.

A total of 2,347 results were identified by the search strategy, from CNKI (327), Wan Fang (672), VPVIP (232), CBM (9), Web of Science (156), PubMed (687), Cochrane Library (70), EBSCO (33), EMBASE (145), APA PsycNet (7), and Scopus (9). After deduplication, 1,743 studies were excluded, followed by 1,342 studies after preliminary screening. After seeking studies screened, five studies not retrieved were excluded. After reading full texts, 14 studies were excluded as missing sample size, 217 studies were excluded as data unavailable, and 110 studies were excluded as no control group. Finally, 39 studies were included in the final analysis (Figure 1). To reconfirm the completeness of included studies for this study, we manually screened reference lists of related published reviews and meta-analyses for additional relevant studies (Wu et al., 2019; Zhang C. et al., 2022). Our careful examination revealed that included studies in this study not only contained all included studies in the relevant published literature but also a significant number of new studies.

### 3.2. Characteristics of the included studies and risk of bias assessment

**Figure 2:**
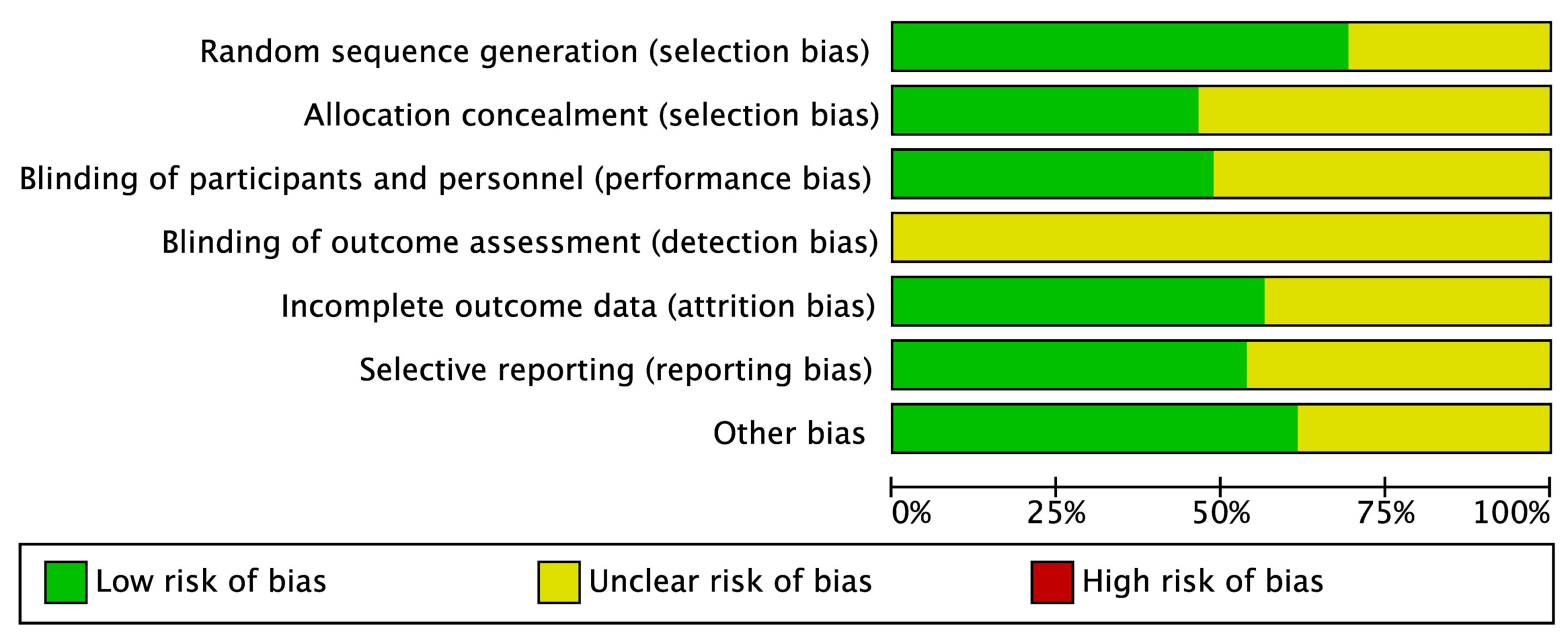
Bias risk of the included studies.

There were 2,408 students with IA being examined in 39 studies. There were six types of exercise interventions (team sport, double sport, single sport, team + double sport, team + single sport, and team + double + single sport), and all interventions in the control group were in a no-intervention manner, and the basic characteristics of the included studies are shown in Table 1. Six studies were a three-armed trial, one study was a four-armed trial, and the others were all two-armed trials. The studies all mentioned randomized grouping and only 27 described the specific randomized sequence method; the remaining studies did not describe the allocation method in detail (Figure 2). The summary of the risk of bias judgments for each included study was presented in the Supplementary File.

**Table 1:** Summary of studies included in network meta-analysis indicating the exercise intervention used and the outcome measure.

**Reference**	**Location (Language)**	**Participant characteristics**			**Experiment group**			**Outcome variables (Assessment tools)**
		**Sample source (Age)**	**Sample size (T/C)**	**Gender (female/male)**	**Exercise program duration (week)**	**Frequency/ Does/Length**	**Intervention type**	
Yang (2021)	Hengyang, China (Chinese)	College student (NR)	28/14	60/24	12	2-3/week Moderate intensity (130-150b/ min) Length NR	Team Sport (Basketball)	Internet addiction (CIAS); Loneliness (UCLA)
			28/14				Single Sport (Running)	
Ren et al. (2014)	Jiamusi, China (Chinese)	College student (20-24)	4/4	6/2	12	3/week Moderate intensity 90-120 min/session	Single Sport (Yoga)	Internet addiction (YIAS): Mental health (SCL-90)
Zhu (2008)	Nanjing, China (Chinese)	College student (20.12)	6/6	NR	12	3-4/week Moderate intensity (100-120b/ min) Length NR	Team Sport (Basketball)	Internet addiction (YIAS); Mental health (SCL-90)
Zhang (2013)	Kun ming, China (Chinese)	College student (NR)	30/30	NR	16	2/week Moderate intensity Length NR	Team + Double Sport (Basketball + Tennis)	Internet addiction (YIAS)
Gao et al. (2012)	Changchun, China (Chinese)	College student (NR)	35/34	36/33	8	5/week Intensity NR 90 min/session	Single Sport (Running)	Internet addiction (YDQ); Mental health (SCL-90)
Liao (2008)	Zhuzhou, China (Chinese)	College student (NR)	8/14	1/11	10	3/week Intensity NR 70 min/session	Single Sport (Dance)	Internet addiction (CIAS); Mental health, Depression, Interpersonal sensitivity and Anxiety (SCL-90)
Deng (2003)	Nanchang, China (Chinese)	College student (NR)	14/14	NR	10	3/week Moderate intensity (VO2 max 50-80%) 50 min/session	Team + Double Sport (Basketball + Bad minton)	Internet addiction (CIAS); Mental health, Depression, Interpersonal sensitivity and Anxiety (SCL-90)
Li et al. (2009)	Shijiazhuang, China (Chinese)	College student (NR)	16/16	0/32	8	3/week Intensity NR 40-60 min/session	Double Sport (Bad minton)	Internet addiction (YIAS); Mental health, Depression, Interpersonal sensitivity and Anxiety (SCL-90)
		**Sample source (Age)**	**Sample size (T/C)**	**Gender (female/male)**	**Exercise program duration (week)**	**Frequency/ Does/Length**	**Intervention type**	
Lou (2011)	Shengyang, China (Chinese)	Middle school	18/18	36/0	12	3/week Intensity NR 90-120 min/session	Team Sport (Basketball)	Internet addiction (YIAS); Mental health, Depression, Interpersonal sensitivity and Anxiety (SCL-90)
Li M. et al. (2014)	Shijiazhuang, China (Chinese)	Middle school student (15.51 ± 1.62)	27/24	51/0	10	3/week High intensity (VO2 max 90%) 60-70 min/session	Single Sport (HIIT)	Internet addiction (YDQ)
Li et al. (2021)	Xiangtan, China (Spanish)	Middle school student (NR)	59/62	58/63	12	5/week Intensity NR 40 min/session	Single Sport (Dance)	Internet addiction (IADDS)
Fu and Liu (2016)	Changchun, China (Chinese)	College student (20.41 ± 1.37)	42/42	34/50	16	3/week Intensity NR 50 min/session	Team Sport (Football)	Internet addiction (YDQ)
Yang and Zeng (2017)	Fuzhou, China (Chinese)	College student (19.65 ± 1.3)	26/26	NR	16	4/week Intensity NR 60 min/session	Single Sport (Tai Chi)	Internet addiction (CIAS)
Zhang (2011)	Shangqiu, China (Chinese)	College student (NR)	18/18	NR	12	3/week Intensity NR 90-120 min/session	Team Sport (Basketball)	Internet addiction (YIAS); Mental health, Depression, Interpersonal sensitivity and Anxiety (SCL-90)
Ji (2017)	Beijing, China (Chinese)	College student (NR)	10/10	11/9	12	3/week Moderate intensity 60 min/session	Team + Single Sport (Basketball + Running)	Internet addiction (YIAS); Mental health (SCL-90)
Fan (2017)	Zhengzhou, China (Chinese)	College student (NR)	15/15	22/8	12	3/week Moderate intensity (130-150b/ min) 60 min/session	Team Sport (Basketball)	Internet addiction (CIAS)
Wang (2016)	Taiyuan, China (Chinese)	College student (NR)	36/37	23/50	12	3/week Intensity NR 45 min/session	Team + Double Sport Sport (Basketball + Table Tennis)	Internet addiction (SAS-C)
Ge et al. (2015)	Nanchang, China (Chinese)	College student (20.13 ± 1.35)	18/18	24/12	18	3/week Low intensity 120 min/session	Team Sport (Volleyball)	Internet addiction (MPAI)
Yang (2021)	Fuzhou, China (Chinese)	College student (NR)	90/15	38.7/61.3	8	2/week Moderate intensity 60 min/session	Single Sport (Running + Tai Chi)	Internet addiction (MPATS)
			90/15				Double Sport (Table tennis + Bad minton)	
Zhang et al. (2015)	Loudi, China (Chinese)	College student (NR)	40/40	54.6/45.4	8	2/week Intensity NR 45 min/session	Team Sport (Group outdoor sport games)	Internet addiction (MPATS)
Liao et al. (2022)	Guangzhou, China (Chinese)	College student (20.12 ± 1.54)	8/4	NR	6	2/week Intensity NR Length NR	Team Sport (Basketball)	Internet addiction (MPAI); Loneliness (UCLA)
			8/4				Single Sport (Running)	
Liu et al. (2022)	Wuhan, China (Chinese)	College student (NR)	31/17	76/20	10	2/week Moderate intensity 60 min/session	Team Sport (Basketball)	Internet addiction (MPAI)
			31/17				Single Sport (Ba Duan Jin)	
Yu and Xie (2010)	Hangzhou, China (Chinese)	College student (18-22)	15/15	30/0	8	3/week Moderate intensity (130-150b/ min) 40-60 min/session	Team + Double + Single Sport (Basketball + Bad minton + Running)	Internet addiction (YIAS); Mental health (SCL-90)
Wang F. (2021)	Guangzhou, China (Chinese)	Middle school student (12-13)	17/16	17/16	9	2/week Moderate intensity 40 min/session	Team Sport (Basketball)	Internet addiction (SAS)
		**Sample source (Age)**	**Sample size (T/C)**	**Gender (female/male)**	**Exercise program duration (week)**	**Frequency/ Does/Length**	**Intervention type**	
Xu (2019)	Fuzhou, China (Chinese)	College student (NR)	22/15	29/31	16	3/week Intensity NR 60 min/session	Single Sport (Running)	Internet addiction (MPATS)
			8/15				Team + Double Sport (Basketball + Bad minton)	
Zhu (2017)	Hangzhou, China (Chinese)	College student (18-23)	30/30	26/34	8	3/week Moderate intensity (130-140b/ min) 60 min/session	Team + Double + Single Sport (Ball games + yoga)	Internet addiction (MPATS)
Wang L. (2021)	Jinan, China (Chinese)	College student (NR)	21/21	20/22	8	3/week Intensity NR Length NR	Team + Single Sport (Volleyball + Dance)	Internet addiction (MPATS)
Bu (2014)	Zhengzhou, China (Chinese)	College student (18-21)	30/30	NR	24	3-5/week Moderate intensity (VO2 max 60-80%) 30-90 min/session	Team + Double + Single Sport (Basketball + Bad minton + Running)	Internet addiction (MPATS)
Zhang (2012)	Beijing, China (Chinese)	College student (20.4)	17/13	NR	10	3/week Moderate intensity (VO2 max 50-80%) Length NR	Team + Double + Single Sport (Ball games + Dance + Running)	Internet addiction (CIAS)
Zhou et al. (2011)	Zhengzhou, China (Chinese)	College student (20.3 ± 1.64)	20/18	20/18	12	3/week Intensity NR 60 min/session	Team + Double + Single Sport (Basketball + Bad minton + Bicycle)	Internet addiction (MPDQ)
Fu and Liu (2010)	Jiujiang, China (Chinese)	Middle school (16.4)	16/16	8/8	12	3/week Moderate intensity (130-150b/ min) 40 min/session	Team + Double + Single Sport (Basketball + Bad minton + Dance)	Internet addiction (YIAS); Mental health (SCL-90)
Yu (2017)	Guangzhou, China (Chinese)	College student (NR)	26/26	40/60	8	2/week Intensity NR 120 min/session	Team + Double + Single Sport (Basketball + Bad minton + Weight lifting)	Internet addiction (YIAS)
		**Sample source (Age)**	**Sample size (T/C)**	**Gender (female/male)**	**Exercise program duration (week)**	**Frequency/ Does/Length**	**Intervention type**	
Yang et al. (2022)	Yangzhou, China (Chinese)	Middle school student (16.28 ± 1.67)	36/18	62/46	12	3/week Moderate intensity (123-140b/ min) 45 min/session	Single Sport (Dance)	Internet addiction (MPAI)
			36/18				Double Sport (Bad minton)	
Liu et al. (2022)	Taiyuan, China (Chinese)	Primary school student (9-12)	70/70	72/68	10	2-4/week Intensity NR 120 min/session	Team + Double Sport (Basketball + Bad minton)	Internet addiction (SAS)
Chi (2012)	Changchun, China (Chinese)	College student (NR)	10/10	11/9	8	5/week Moderate intensity (130-150b/ min) 90 min/session	Single Sport (Running + Bicycle)	Internet addiction (YIAS)
Liu et al. (2019)	Wuhan, China (Chinese)	College student (19.29 ± 1.22)	31/17	70/26	12	3/week Intensity NR 90 min/session	Team Sport (Basketball)	Internet addiction (MPAI); Mental health (SCL-90)
			31/17				Single Sport (Ba Duan Jin)	
Fan et al. (2021)	Jurong, China (Chinese)	College student (20.13 ± 1.02)	32/32	32/32	14	3/week Intensity NR 90 min/session	Team + Double + Single Sport (Volleyball + Table tennis + Dance)	Internet addiction (SAS-C)
Xiao et al. (2021)	Shenzhen, China (English)	College student (19.08 ± 1.22)	31/17	71/25	12	3/week Moderate intensity (120-150b/ min) 90 min/session	Team Sport (Basketball)	Internet addiction (MPAI); Loneliness (UCLA); Anxiety (SRAS)
			31/17				Single Sport (Ba Duan Jin)	
Zheng et al. (2019)	Yiyang, China (Chinese)	College student (17-20)	50/49	NR	8	5/week Intensity NR 60 min/session	Team Sport (Basketball)	Internet addiction (AMPUD)

T, treatment group; C, control group; YIAS, Comprehensive Internet Addiction Diagnostic Questionnaire; YDQ, Internet Addiction Damage Scale; CIAS, Chinese Internet Addiction Scale; SAS-C, Smartphone Addiction Scale for College Students; MPAI, Mobile Phone Addiction Inventory Scale. MPATS, Mobile Phone Dependence Scale for College Students; IADDS, Internet Addiction Deter mination Scale for Secondary School Students; MPDQ, Mobile Phone Dependence Questionnaire for College Students; AMPUD, Self-Assessment Questionnaire for Adolescent Mobile Phone Use Dependence; SAS, Smartphone-Based on Diagnostic Criteria for Internet Addiction; UCLA, UCLA Loneliness Scale; SCL-90, 90-item Symptom Checklist 90; SRAS, Self-Assessment Anxiety Scale. ① Internet addiction; ② Mental health; ③ Loneliness; ④ Depression; ⑤ Interpersonal sensitivity; ⑥ Anxiety.

### 3.3. Meta-analysis

Due to the number of included studies in indicators (loneliness, anxiety, depression, and interpersonal sensitivity) being insufficient to conduct a network meta-analysis, only a meta-analysis was performed. The detailed data of the meta-analysis are in the Supplementary material. The results of the meta-analysis showed that I^2^ = 77.8%, for loneliness; I^2^ = 81.2%, for anxiety; I^2^ = 75.7%, for depression; I^2^ = 77.5%, for interpersonal sensitivity, so we only report the random-effects results.

#### 3.3.1. Loneliness

To test H1a that IA students in exercise intervention have a positive effect on loneliness, four studies with 478 subjects were included in this study. The result showed that exercise in IA students had a significant effect on improving loneliness compared to no intervention (SMD = −0.96, 95% CI −1.5 to −0.41, *p* < 0.05).

#### 3.3.2. Anxiety

To test H1b that IA students in exercise intervention have a positive effect on anxiety, seven studies with 377 subjects were included in this study. The study showed that exercise in IA students had a significant effect on improving anxiety compared to no intervention (SMD = −1.79, 95% CI −2.37 to −1.22, *p* < 0.05).

#### 3.3.3. Depression

To test H1c that IA students in exercise intervention have a positive effect on depression, five studies with 182 subjects were included in this study. The results showed that exercise in IA students had a significant effect on improving depression compared to no intervention (SMD = −1.5, 95% CI −2.19 to −0.81, *p* < 0.05).

#### 3.3.4. Interpersonal sensitivity

To test H1d that IA students in exercise intervention have a positive effect on interpersonal sensitivity, five studies with 182 subjects were included in this study. The study showed that exercise in IA students had a significant effect on improving interpersonal sensitivity compared to no intervention (SMD = −1.34, 95% CI −2.05 to −0.64, *p* < 0.05).

### 3.4. Network meta-analysis

**Figure 3:**
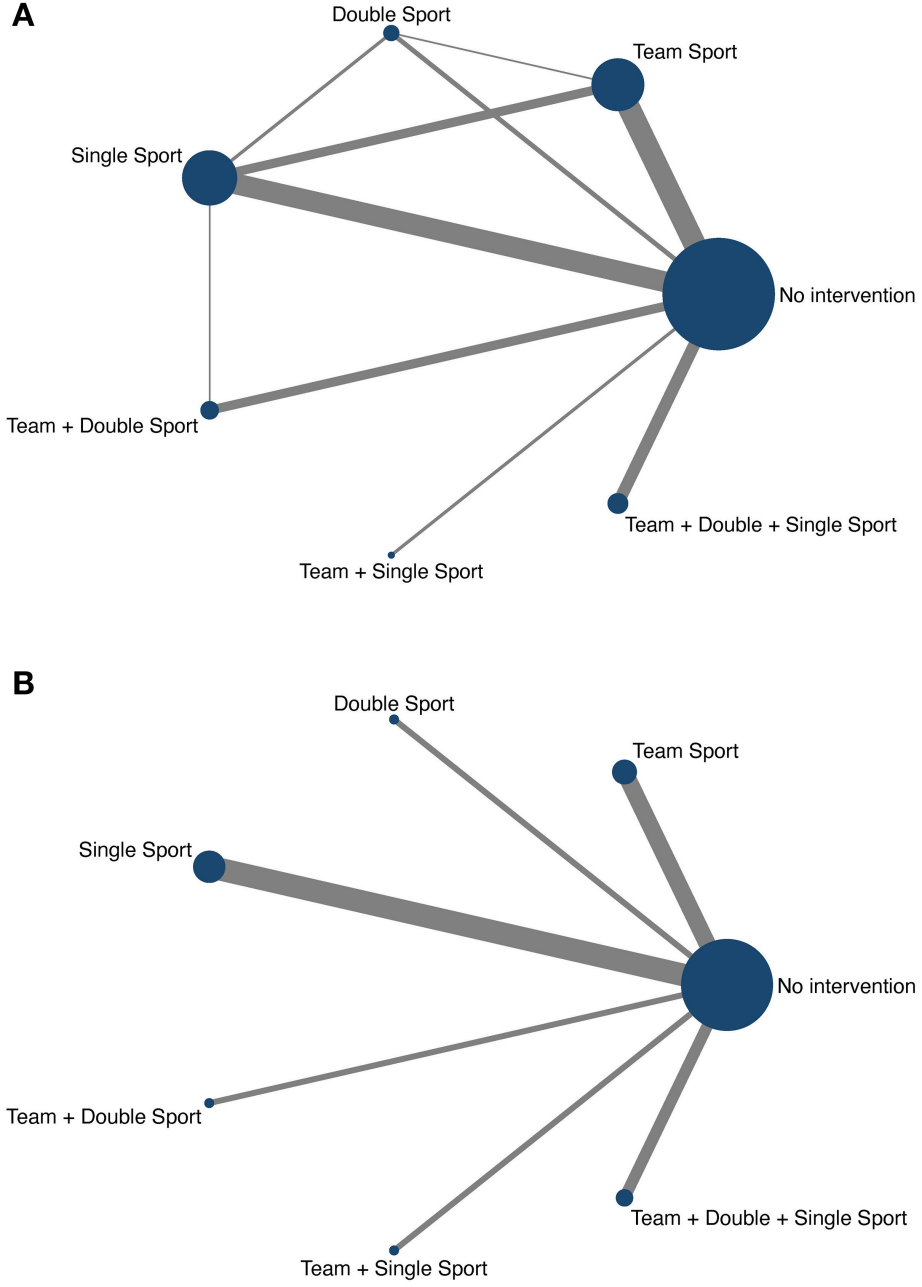
Network meta-analysis maps of the studies examining the efficacy of exercise interventions on **(A)** Internet addiction and **(B)** Mental health.

To test H2 that the double sport with some interaction has the best intervention effects on IA students for Internet addiction (H2a) and mental health (H2b), 39 studies were included in the network meta-analysis to examine the improvement of exercise for IA (Figure 3A), and 12 studies were included to examine the benefits for mental health (Figure 3B), both including six exercise intervention types and one no intervention. In the network meta-analysis map, each node represents an intervention type, and the connecting lines indicated studies with direct comparisons between intervention types. The thickness of the connecting lines correlates with the number of studies between measures, while the size of the nodes was proportional to the number of studies per node.

#### 3.4.1. Internet addiction

This network map showed closed loops, and the consistency of each closed loop needs to be further evaluated (Figure 3A). The inconsistency test of the results of each closed loop using nodal analysis showed that the values of the inconsistency factors ranged from 0.483 to 1.827, and the lower limit of 95% CI was 0. The consistency of each closed loop was good, indicating that the constituted network relationship map met the consistency assumption.

The results of the two-by-two comparison are shown in Table 2. Compared with the no-intervention team, the team sport, the double sport, the single sport, the team + double sport, and the team + double + single sport in reducing IA were statistically significantly different. In the comparison of different exercise types, the double sport, the team + double + single sport, and the team + double sport had a better effect than the single sport in reducing IA, and the differences between other comparisons were not statistically significant.

**Table 2:** League table for head–to–head comparisons.

**Double Sport**	−84.68^*^(−143.21, −26.16)	**−98.44 (−166.87, 30.01)**	**−13.72(−72.19, 44.76)**	**−59.74 (−132.23, 12.75)**	**−53.23(−108.62, 2.16)**	−107.1^*^ (−155.54, −58.66)
−0.27 (−2.13, 1.59)	Team + Double + Single Sport	−13.76 (−72.19, 44.68)	−70.97^*^(−117.64, −24.29)	−24.94 (−88.09, 38.20)	−31.45(−74.00, 11.09)	−22.42 (−55.26, 10.43)
−0.30 (−2.22, 1.61)	−0.03 (−1.61, 1.55)	Team + Double Sport	−84.72^*^ (−143.11, −26.34)	−38.70 (−111.12, 33.72)	−45.21 (−100.50, 10.08)	−8.66 (−56.99, 39.67)
−1.51 (−3.13, 0.12)	−1.24 (−2.52, 0.05)	−1.20 (−2.58, 0.17)	Team Sport	−46.02 (−109.12, 17.08)	−39.51 (−80.83, 1.80)	−93.38^*^ (−126.14, −60.63)
−2.06 (−4.65, 0.53)	−1.79 (−4.14, 0.56)	−1.76 (−4.16, 0.64)	−0.55 (−2.77, 1.66)	Team + Single Sport	−0.14 (−2.36, 2.08)	−47.36 (−101.29, 6.57)
−1.92^*^ (−3.50, −0.34)	−1.65^*^ (−2.95, −0.35)	−1.62^*^ (−2.97, −0.27)	−0.41 (−1.32, 0.49)	0.14 (−2.08, 2.36)	Single Sport	−53.87^*^ (−80.73, −27.01)
−3.42 ^*^ (−4.94, −1.89)	−3.14^*^ (−4.20, −2.08)	−3.11^*^ (−4.29, −1.94)	−1.91^*^ (−2.64, −1.18)	−1.35 (−3.45, 0.74)	−1.49^*^ (−2.24, −0.75)	No intervention

^*^p < 0.05. Mental health (upper) and Internet addiction (lower) are reported as Hedges' g and 95% confidence intervals. Negative scores indicate a greater decrease in depressive symptoms for the column group.

**Figure 4:**
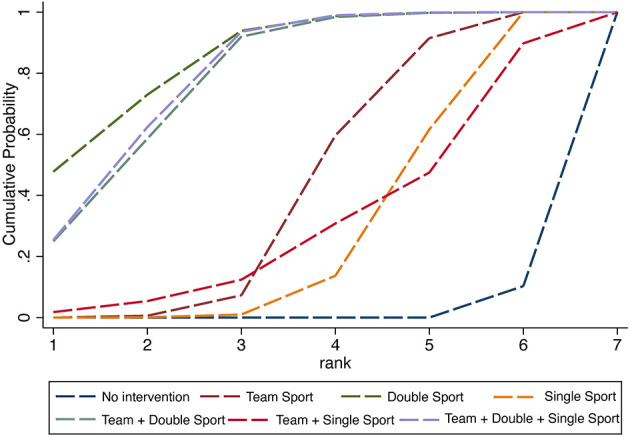
The SUCRA ranking chart for internet addiction.

According to the SUCRA method, the effectiveness of different exercise types in reducing IA was ranked. A SUCRA graph of the effectiveness of interventions was formed and is shown in Figure 4. The results showed that the rank of the effectiveness of different exercise types was double sport (SUCRA = 85.5) > team + double + single sport (SUCRA = 80) > team + double sport (SUCRA = 78.9) > team sport (SUCRA = 43.2) > team + single sport (SUCRA = 31.3) > single sport (SUCRA = 29.4) > no intervention (SUCRA = 1.7).

#### 3.4.2. Mental health

This network map has no closed loops, so there is no need to further evaluate the inconsistency. The results of the pairwise comparison are shown in Table 2. Compared to the no intervention, the differences were statistically significant for the team sport, the double sport, and the single sport in improving the mental health of IA students. In the pairwise comparison of different exercise types, the double sport was a more significant effect than the team + double + single sport and the team + double sport. The differences between the other comparisons were not statistically significant.

**Figure 5:**
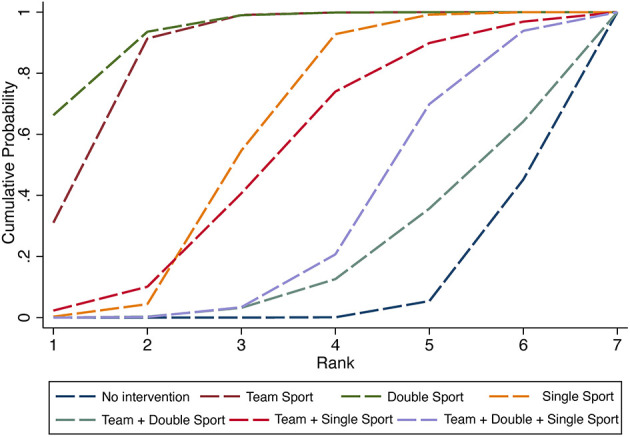
The SUCRA ranking chart for mental health.

According to the SUCRA method, the effectiveness of different exercise types in IA students' mental health was ranked in Figure 5. The results showed that the rank of the effectiveness of different exercise types was double sport (SUCRA = 93.1) > team sport (SUCRA = 86.9) > single sport (SUCRA = 58.6) > team + single sport (SUCRA = 52.3) > team + double + single sport (SUCRA = 31.3) > team + double sport (SUCRA = 19.3) > no intervention (SUCRA = 8.4).

#### 3.4.3. Internet addiction vs. mental health

**Figure 6:**
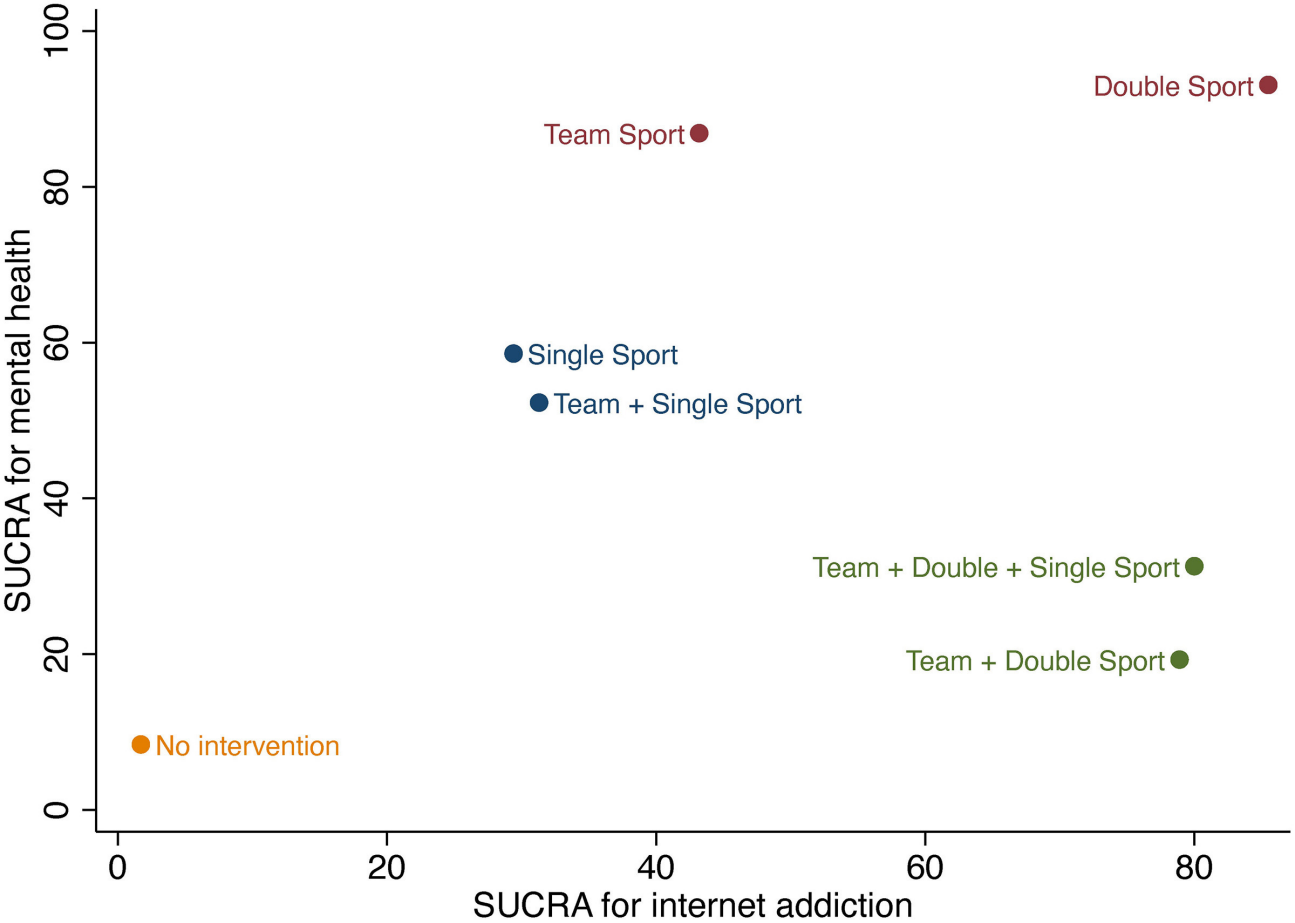
Clustered ranking plot of the network presenting clustered analysis of SUCRA values for effectiveness of internet addiction and mental health.

To test H3 that the double sport is the optimal exercise type under the combined consideration of IA and mental health, a two-dimensional clustered ranking map was performed to show the comprehensive superiority of different exercise types in improving Internet addiction and mental health (Figure 6). According to the analysis, the double sport showed the greatest potential to be the most effective therapeutic exercise type for reducing IA and improving mental health (cluster ranking value: 3699.73).

### 3.5. Publication bias and sensitivity analysis

We assessed the publication bias of this research of every outcome by funnel plots. The horizontal coordinate is the effect size, and the vertical coordinate is the standard error. The studies were evenly distributed on both sides of the midline and were more symmetrical in distribution, but some fell outside the funnel, indicating possible publication bias and small sample effects. Simultaneously, the data remained unchanged following the sensitivity analysis, indicating that the results of this study were stable. Thus, the current analysis results were regarded as reliable (see Supplementary material).

## 4. Discussion

This is the first network meta-analysis of the improvement effect of exercise interventions on IA students. A total of 39 RCTs of exercise interventions and 2,408 IA students were included in the network meta-analysis, our study compensated for the lack of sample size in Internet addiction research. The results from our meta-analysis confirmed the importance of exercise in the intervention of IA students. Specifically, the results showed that single sport, team sport, double sport, team + double sport, and team + double + single sport all significantly reduced IA compared with the control group. In terms of improving mental health in IA students, the single sport, team sport, and double sport produced a significant positive effect. Interestingly, the double sport showed the greatest potential to be the best choice for reducing IA and improving mental health. Taken together, these findings provided robust evidence that exercise is effective in reducing IA in students, with the double sport being the most effective.

Our study found that team sport, double sport, and single sport are significantly effective compared to the control group in both reducing IA and improving mental health for IA students by the network meta-analysis. One very important argument for exercise's beneficial effect on IA is that exercise significantly improved mental health. Recent research has suggested that the scores of loneliness, anxiety, depression, and interpersonal sensitivity of IA students were significantly higher than those of ordinary students (Long et al., 2021; Zhang et al., 2021; Qiu et al., 2022; Yang et al., 2022). Specifically, students with high interpersonal sensitivity have often suffered from loneliness, anxiety, and depression, and progressively increased engagement in the Internet that offered emotional support and expression (Young, 1996; Ang et al., 2012; Gámez-Guadix et al., 2012). It has been reported that exercise can relieve IA by decreasing interpersonal sensitivity, loneliness, anxiety, and depression (Van der Aa et al., 2009; Kheyrkhah et al., 2010). Meanwhile, it has also been found that self-efficacy also improves IA through improved mental health (Cao et al., 2010). However, these studies all suffer from the problem of a small sample size. Instead, we addressed this issue by a meta-analysis, based on a large number of studies and large samples. Our study identified positive effects of exercise interventions on psychological factors related to IA students, that is, exercise interventions improved loneliness, anxiety, depression, and interpersonal sensitivity. Taken together, given that exercise can reduce IA by improving interpersonal sensitivity, loneliness, anxiety, and depression in IA students, it would be appropriate to determine whether exercise intervention is effective. Our study bridges a gap in the lack of a large sample in studies for IA students and conducts an innovative use of network meta-analysis to further examine the intervention effects of different exercise types. From the discussion above, mental health is an important factor in IA (Ko et al., 2012; Babadi-Akashe et al., 2014), thus treatment should not only focus on whether IA is reduced but also on the improvement of mental health (Li et al., 2017; Zhang et al., 2018).

Exercise-improving IA has been revealed in previous studies by meta-analysis (Wu et al., 2019), but whether differences in the intervention effect existed between different exercise types are unknown. The most interesting finding is that the double sport was the most effective exercise type for IA students, based on the effect of combinedly reducing IA and improving mental health by the two-dimensional clustered ranking map of the network meta-analysis. This finding is contrary to previous studies which have suggested that team sport is the best exercise type (Liu, 2013). Their result was explained by the relationship between the stimulation of social support and individual mental health. Social support refers to the influence that individuals obtain through social contacts that can reduce psychological stress, and relieve mental tension (Barrera Jr and Ainlay, 1983). But our research argues that the double sport not only gains social support but also enables addicts to increase the blood flow in the body with continuous double sport exercise. This way causes a certain good stimulation of the central nervous system of the body and promotes the formation of positive emotions in IA students and achieves the purpose of reducing IA (Fu and Liu, 2016; Yang et al., 2016; Shi et al., 2017). Meanwhile, the double sport can bring more stimulation and pleasure generated by competition. It can satisfy the human body's pursuit of changing, novel, and complex sensations and experiences and produce more effective intervention effects (Deng, 2003; Liu et al., 2010; Ma, 2010; Wang, 2012; Hu and Zhang, 2016). It can, therefore, be assumed that double sport should be the main type when doctors or teachers choose a suitable exercise type for IA students. Our study bridges a gap in the lack of studies for seeking the best effective exercise type in IA students and conducts an innovative use of the two-dimensional clustered ranking map to further examine the comprehensive effects of different exercise types in IA and related mental factors.

### 4.1. Practical implications

With consideration of the projected growth of IA students and the consequential intervention demand, the findings from this network meta-analysis offer support for exercise prescription to reduce IA in students. IA damages students' physical and mental health and triggers a series of serious consequences, which is an urgent problem for families, schools, and society (Beutel et al., 2011; Zhou et al., 2011; Li et al., 2015). Thus, our study has practical implications for society, schools, and families in dealing with IA in students. This study concluded that double sport is the best effective exercise type for IA students. Exercise has shown superiority and significance as a low-cost, easily disseminated, and highly adherent intervention for the treatment of substance addiction and behavioral addiction (Bu et al., 2010). Studies found that exercise can promote the adaptive remodeling of reward circuits and reduce recurrent Internet-addictive behaviors in adolescents (Ma et al., 2022b). On the one hand, exercise can activate the same reward circuits as IA, causing adolescents to feel euphoria and satisfaction similar to Internet use, effectively reducing the positive reinforcement of addiction sources (Liu and Wang, 2020). On the other hand, high dopamine levels underlie psychiatric dependence and addiction relapse (Wang et al., 2011), and exercise can reduce dopamine release and receptor utilization, inhibiting adolescents' reward expectations (Zhao et al., 2018). Taken together, schools, as the main place where most students conduct their social life have the mission and responsibility to intervene in the IA problem. Schools should make full use of their teachers and facilities to cultivate students' interest in exercise to reduce the occurrence of IA behaviors. Meanwhile, family and society should regulate youth behavior and eventually correct IA effectively by cultivating their interest in exercise, establishing the concept of healthy living, and creating a good family relationship and growth environment.

### 4.2. Limitation

The current network meta-analysis is not without limitations. First, the results of the meta-analysis on loneliness, anxiety, depression, and interpersonal sensitivity showed significant heterogeneity. This was due to the small number of included studies. Therefore, we conducted sensitivity analyses through screened-out one-by-one included studies and found that none of the data changed substantially, indicating some stability in the results of this study. Second, we only compared different exercise types and did not compare the exercise intensity and exercise strength. It is because most of the included studies used the same intensity and had no differences to compare. The included studies were different in terms of exercise duration, but we found no common classification in duration, so only the exercise type classification was used for comparison in this study. However, we conducted regression analyses for frequency, length of a session, duration, and length of a week on the effect of reducing IA, and the results of the study showed no significant differences (see the Supplementary material). Third, most of the included studies in this study were self-reported by scales. The term “addiction” may be inaccurate when used without a diagnosis by a psychiatrist. But scale-based self-reports can reduce interviewers' prejudices, are anonymous, allow thoughtful answers instead of immediate responses, and can be used for a wider range of samples to support us in solving urgent problems (Kuss et al., 2014). Studies on other psychological problems found no significant differences between self-reported and clinical assessments (Polaino and Senra, 1991). However, we suggest that the study findings should be viewed with caution. Fourth, the included study in this study includes mobile phone addiction. Internet addiction and mobile phone addiction are quite similar, both are behavioral addictions (Kwon et al., 2013; Mok et al., 2014) with similar attributes and characteristics (Chen et al., 2022), and similar items of measurement instruments are used (Chi and Chiu, 2013; Kuss et al., 2014; Hadlington, 2015). We believe that the smartphone, as a means of accessing the internet, can lead to addictive behavior related to Internet use. But we still need to clarify the distinction that was thought to exist in previous studies. Smartphone addiction has unique characteristics that distinguish it from Internet addiction in terms of behavioral patterns, addiction groups and characteristics (Petry and O'Brien, 2013; Mok et al., 2014; Jeong, 2016), and addiction possibilities (Salehan and Negahban, 2013). However, we suggest that the study findings should be viewed with caution.

### 4.3. Future direction

First, further research is still needed to focus on influence factors in IA, such as loneliness, anxiety, depression, and interpersonal sensitivity. Second, exercise intensity homogeneity should be avoided in future studies, which is beneficial to explore the effects of different intensities in IA. Third, exercise intervention studies in AI students diagnosed by clinician-administered interviews are still needed in further. Fourth, a unified classification of exercise duration should be established in the future to better investigate the effects of different duration in AI. Fifth, the mechanisms of IA should be explored in the future and thus clearly distinguish the boundaries between IA and related addiction problems.

## Data availability statement

The original contributions presented in the study are included in the article/Supplementary material, further inquiries can be directed to the corresponding author.

## Author contributions

YZ conceived the study design and drafted the manuscript. GL and CL participated in data collection and analysis. ZS, HC, and JG conceived the study design, assisted in revising the manuscript, and reviewed the first and final versions of the manuscript. All authors contributed to the manuscript and agreed to the submitted version of the manuscript.
