# Supplementary material for: Mixed comparison of interventions for different exercise types on students with Internet addiction: a network meta-analysis
Source: Front Psychol. 2023 May 25;14:1111195. doi: 10.3389/fpsyg.2023.1111195 (PMC10249056; doi:10.3389/fpsyg.2023.1111195)
Supplement: Supplementary file 1 [file Data_Sheet_1.docx]

Supplementary Material

A mixed comparison of interventions for different exercise types on students with internet addiction: A network meta-analysis

# Supplementary Figures and Tables

**Supplemental figure 1.** Risk of bias in individual studies. +, low risk of bias; ?, unclear risk of bias; –, high risk of bias.

**Supplemental figure 2.** Forest plots for the loneliness.

**Supplemental figure 3.** Forest plots for the anxiety.

**Supplemental figure 4.** Forest plots for the depression.

**Supplemental figure 5.** Forest plots for the interpersonal sensitivity.

**Supplemental figure 6.** Funnel plots for main analysis. (1) Internet addiction. (2) Mental health. (3) loneliness. (4) anxiety. (5) depression. (6) interpersonal sensitivity. (No intervention; B: Team sport; C: Double sport; D:Single sport; E: Team + Double sport; F: Team + Single sport; G: Team + Double + Single sport)

**Supplemental figure 7.** sensitivity analysis. (1) loneliness. (2) anxiety. (3) depression. (4) interpersonal sensitivity.

**Supplemental Table 1.** Search strategy

**Supplemental Table 2.**  Meta-regression analyses.

## Supplementary Figures

**Supplemental figure 1.** Risk of bias in individual studies. +, low risk of bias; ?, unclear risk of bias; –, high risk of bias.


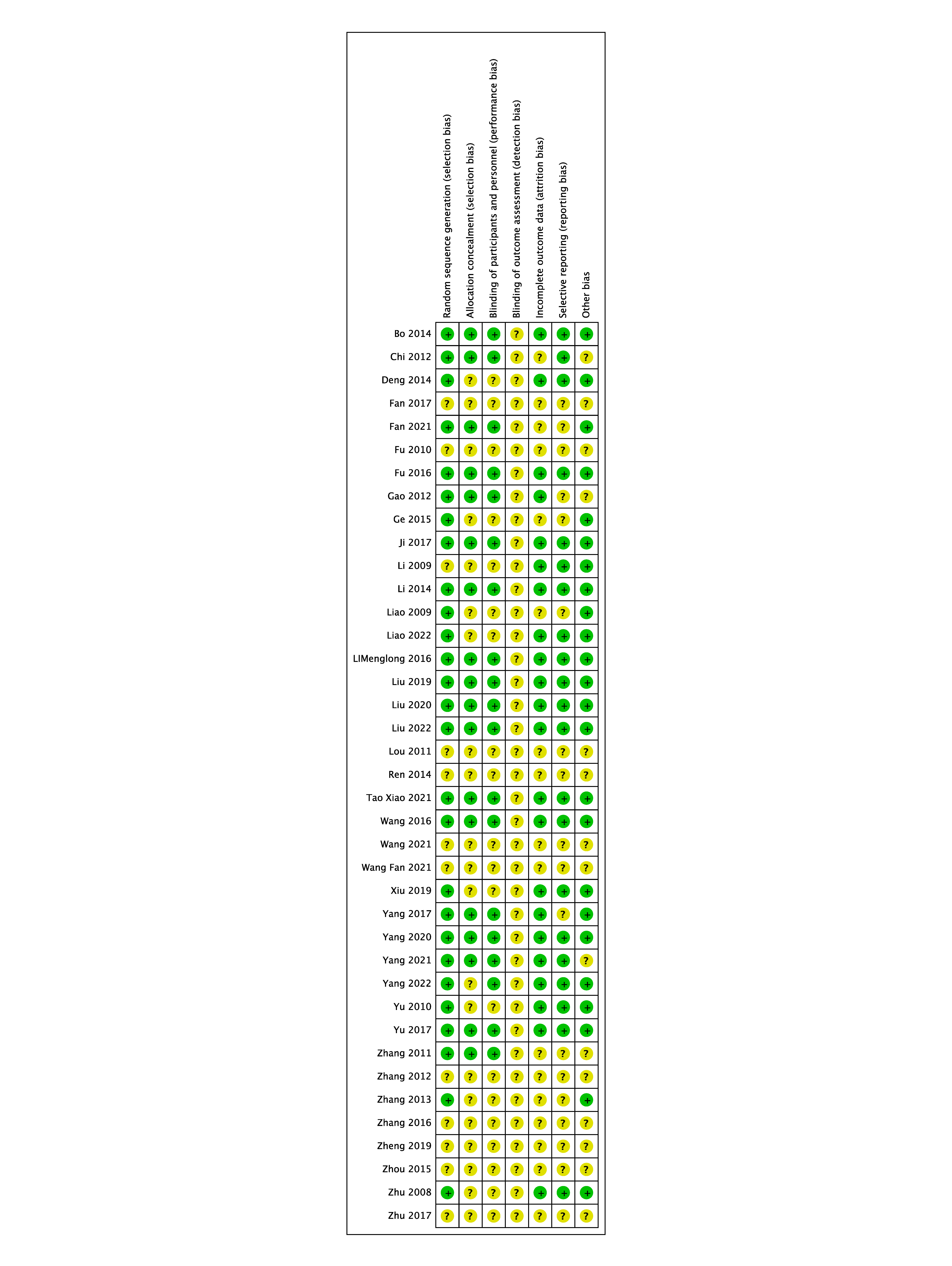


**Supplemental figure 2.** Forest plots for the loneliness.

**
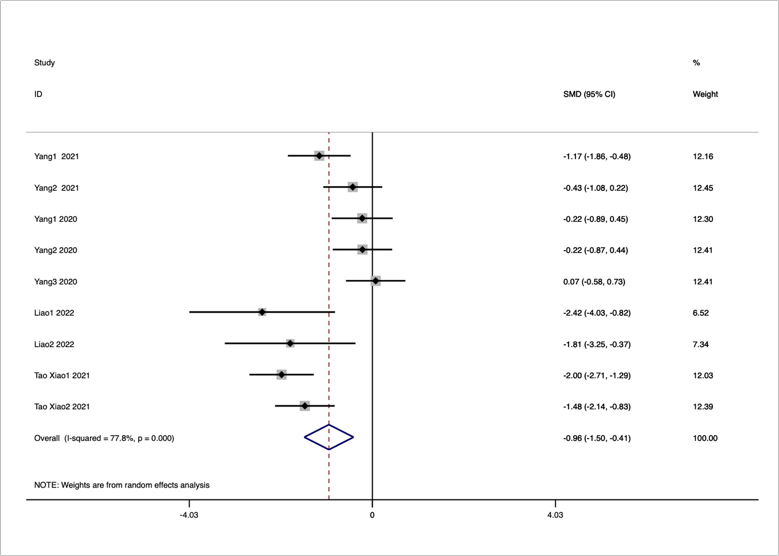
**

**Supplemental figure 3.** Forest plots for anxiety.


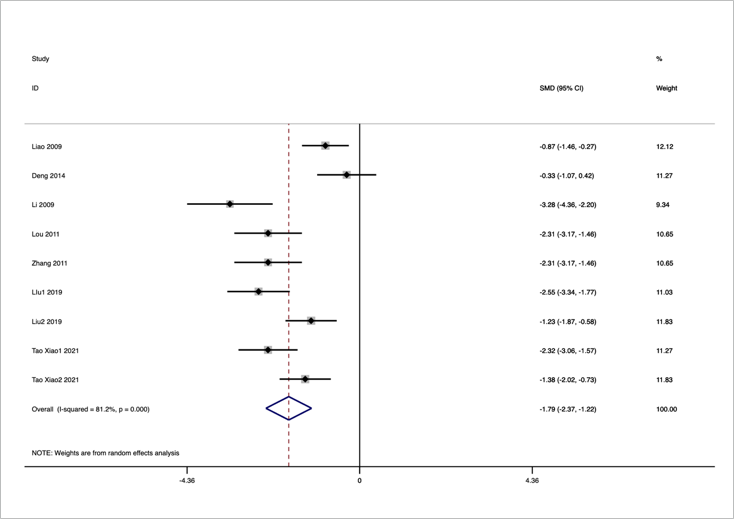


**Supplemental figure 4.** Forest plots for depression.


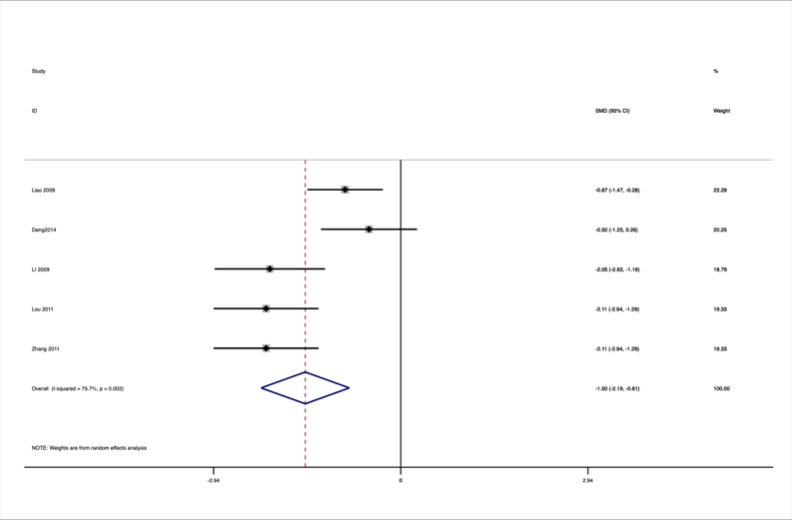


**Supplemental figure 5.** Forest plots for interpersonal sensitivity.


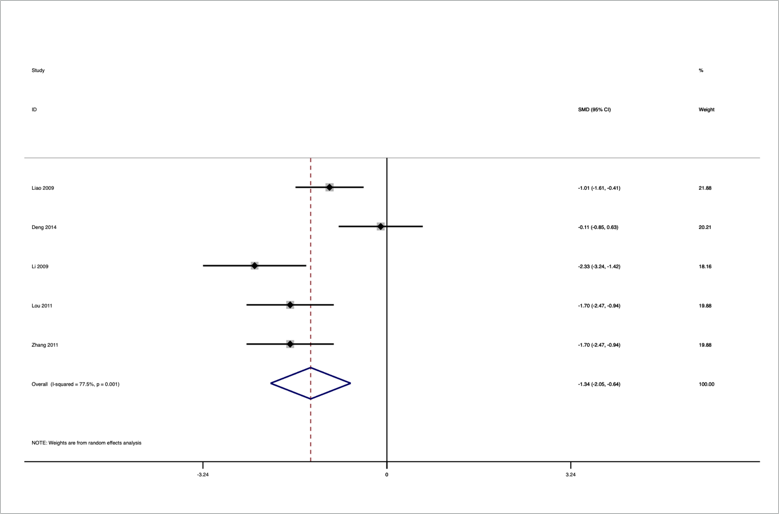


**Supplemental figure 6.** Funnel plots for main analysis. (1) Internet addiction. (2) Mental health. (3) loneliness. (4) anxiety. (5) depression. (6) interpersonal sensitivity.


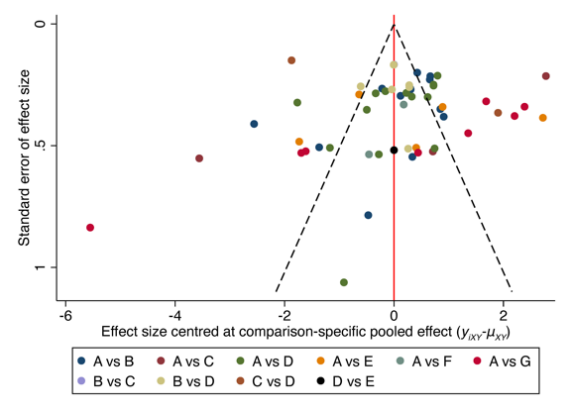

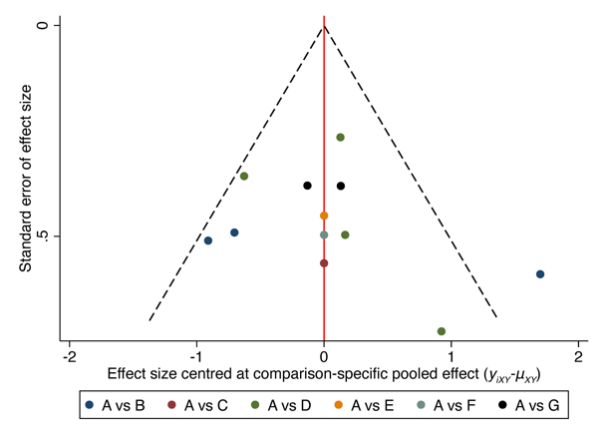


(1) (2)


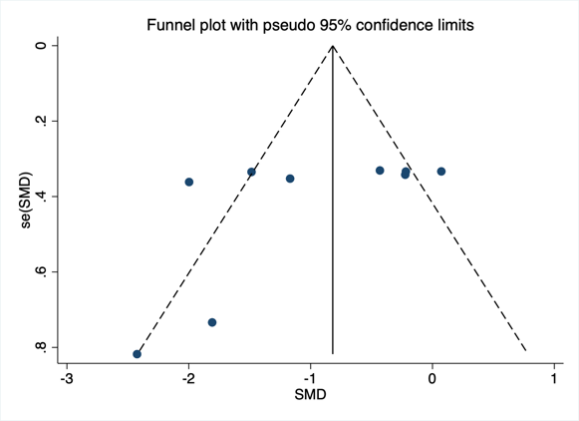

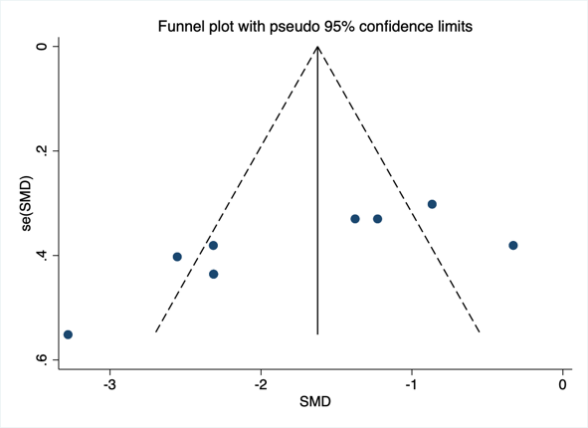


(3) (4)


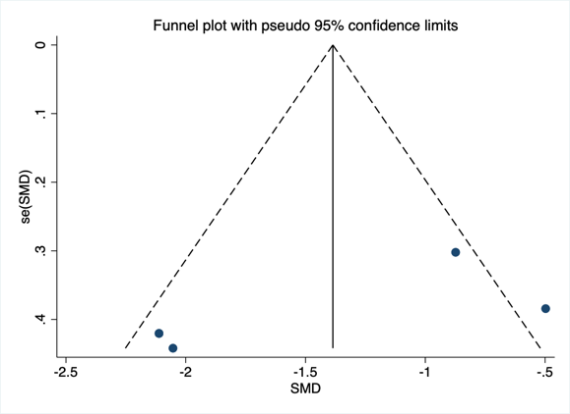

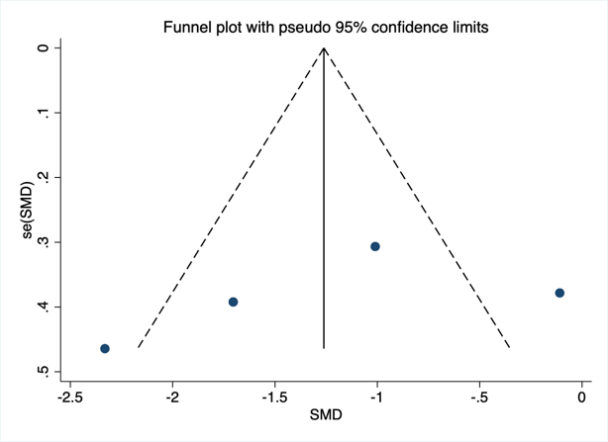


(5) (6)

Note. A: No intervention; B: Team sport; C: Double sport; D:Single sport; E: Team + Double sport; F: Team + Single sport; G: Team + Double + Single sport.

**Supplemental figure 7.** sensitivity analysis. (1) loneliness. (2) anxiety. (3) depression. (4) interpersonal sensitivity.


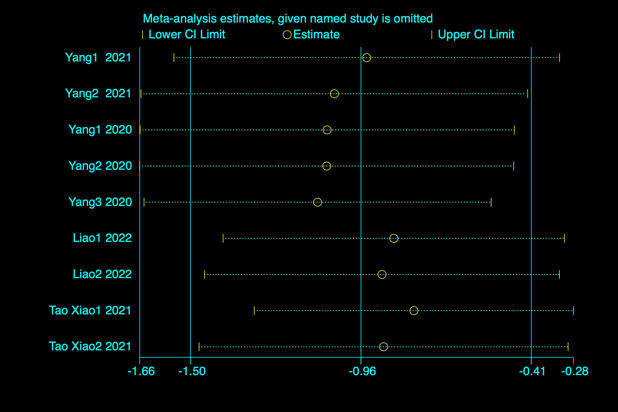


(1)


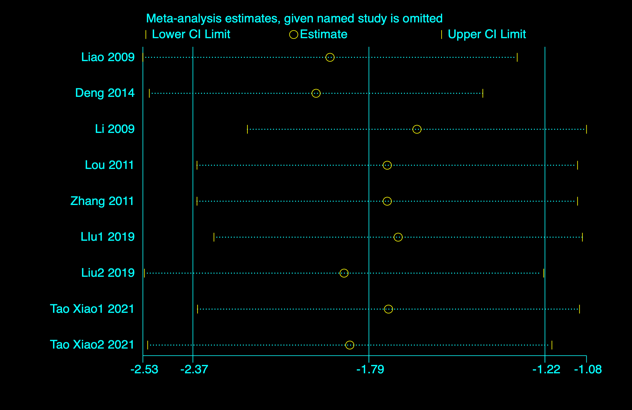


(2)


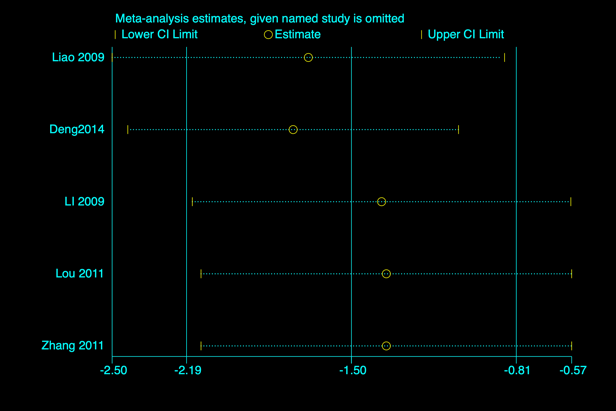


(3)


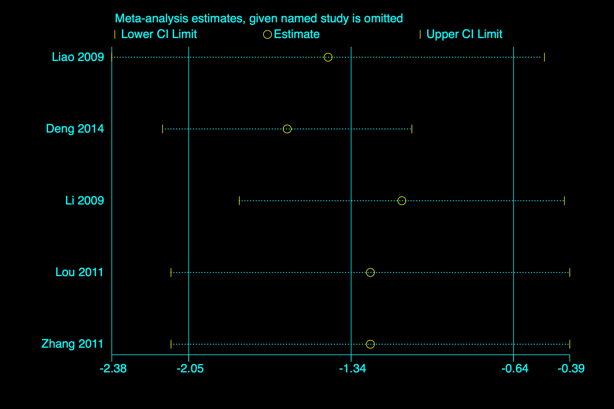


(4)

## Supplementary Table

(“Internet addiction” OR “Addiction Disorder, Internet” OR “Addiction Disorders, Internet” OR “Disorder, Internet Addiction” OR “Disorders, Internet Addiction” OR “Internet Addiction Disorders” OR “Internet Addiction” OR “Addiction, Internet” OR “Addictions, Internet” OR “Internet Addictions” OR “Social Media Addiction” OR “Addiction, Social Media” OR “Addictions, Social Media” OR “Media Addiction, Social” OR “Media Addictions, Social” OR “Social Media Addictions” OR “Smartphone Addiction” OR “Addiction, Smartphone” OR “Addictions, Smartphone” OR “Smartphone Addictions” OR “Internet Gaming Disorder” OR “Disorder, Internet Gaming” OR “Disorders, Internet Gaming” OR “Gaming Disorder, Internet” OR “Gaming Disorders, Internet” OR “Internet Gaming Disorders”) AND (Exercises OR “Physical Activity” OR “Activities, Physical” OR “Activity, Physical” OR “Physical Activities” OR “Exercise, Physical” OR “Exercises, Physical” OR “Physical Exercise” OR “Physical Exercises” OR “Acute Exercise” OR “Acute Exercises” OR “Exercise, Acute” OR “Exercises, Acute” OR “Exercise, Isometric” OR “Exercises, Isometric” OR “Isometric Exercises” OR “Isometric Exercise” OR “Exercise, Aerobic” OR “Aerobic Exercise” OR “Aerobic Exercises” OR “Exercises, Aerobic” OR “Exercise Training” OR “Exercise Trainings” OR “Training, Exercise” OR “Trainings, Exercise”) AND (undergraduates OR “college students” OR youth OR children OR adolescents OR teens OR teenagers OR “young people” OR school OR students OR kid OR pediatrics OR young-adult OR “Adult Students” OR “College Freshmen” OR “First Generation College Student”)

**Supplemental Table 1.** Search strategy.

| _ES | Coef. | Std.Err | t | P>｜t｜ | [95% Conf. Interval] | |
| --- | --- | --- | --- | --- | --- | --- |
| frequency | 0.2934456 | 0.4664246 | 0.63 | 0.534 | -0.6555023 | 1.242393 |
| Length of a session | 0.0128702 | 0.0227193 | 0.57 | 0.575 | -0.0333526 | 0.059093 |
| duration | -0.0918542 | 0.0841124 | -1.09 | 0.283 | -0.2629821 | 0.0792737 |
| length of a week | -0.0025325 | 0.004873 | -0.52 | 0.607 | -0.0124467 | 0.0073818 |
| _cons | -2.404832 | 2.044921 | -1.18 | 0.248 | -6.565256 | 1.755591 |

**Supplemental Table 2.**  Meta-regression analyses.
